# Identification and bioinformatic analysis of CircRNAs in the plasma of patients with very severe chronic obstructive pulmonary disease

**DOI:** 10.1186/s12890-023-02513-5

**Published:** 2023-06-16

**Authors:** Sihui Tang, Yichuan Ding, Zihan Zhou, Wanchun Yang

**Affiliations:** 1 Department of Respiratory and Critical Care Medicine, Hefei Hospital Affiliated to Anhui Medical University, The Second People’s Hospital of Hefei, Hefei, Anhui 230011 China; 2 The Fifth Clinical College of Anhui Medical University, Hefei, Anhui 230032 China; 3 Department of Respiratory and Critical Care Medicine, The Second People’s Hospital of Hefei Affiliated to Bengbu Medical University, Bengbu, Anhui 230030 China

**Keywords:** Chronic obstructive Pulmonary Disease, CircRNA, Microarray, Competing endogenous RNA

## Abstract

**Background:**

The differential expression of circular RNAs (circRNAs) in individuals with very severe chronic obstructive pulmonary disease (COPD) and healthy individuals was screened using microarray technology. The related functions and mechanisms were analyzed using bioinformatic methods to explore the potential of target circRNAs as biomarkers of COPD and provide insights for future pathogenesis.

**Patients and methods:**

Thirty patients with very severe COPD and thirty healthy controls were diagnosed at The Second People’s Hospital of Hefei from September 2021 to September 2022. The differential expression of circRNAs was compared and analyzed using a gene microarray and verified using quantitative real-time polymerase chain reaction (qRT-PCR) technology.

**Results:**

A total of 90 upregulated and 29 downregulated circRNAs were screened in patients with very severe COPD and compared with those in healthy controls. qRT-PCR analysis showed that hsa_circ_0062683 of patients with very severe COPD was significantly upregulated, and hsa_circ_0089763 and hsa_circ_0008882 were significantly downregulated. By constructing the circRNA-miRNA interaction network, it was found that hsa-miR-612, hsa-miR-593-5p, hsa-miR-765, and hsa-miR-103a-2-5p are the miRNAs regulated by more differentially expressed circRNAs (DEcircRNAs). DEcircRNAs may participate in the development of COPD through hypoxia or regulation of various immune cells.

**Conclusion:**

Plasma circRNAs may play a helpful role in the diagnosis and assessment of COPD and be valuable disease biomarkers.

## Background

Chronic obstructive pulmonary disease (COPD) is a lifelong disease characterized by persistent airflow obstruction and respiratory symptoms. As a common disease of the respiratory system, it is the leading cause of death and disability worldwide [1, 2]. As a result of various factors including the aging of the population, the prevalence of COPD will continue to increase in the future and may become the third leading cause of death worldwide in 2030 [3, 4]. In China, the prevalence of COPD varies significantly between men and women, probably due to differences in smoking [5]. As a heterogeneous disease, the treatment of COPD should be guided according to the severity of lung injury, symptoms such as clinical breathing difficulties, and the frequency of exacerbations [6]. In clinical work, patients with very severe COPD have a worse prognosis than patients with mild airflow limitation. Some studies have shown that when compared with moderate and severe groups, patients with very severe COPD experience more obvious discomfort, such as pain and sleep difficulties [7]. Relevant indicators that differentiate patients with very severe COPD from patients may facilitate the development of more personalized treatment plans that can be quickly initiated and allow for early diagnosis, disease assessment, clinical treatment, and disease prognosis.

Circular RNA (circRNA) is an endogenous non-coding RNA that can remain relatively stable in the plasma. Its biogenesis is regulated by specific cis-acting elements and trans-acting factors [8]. Structurally, circRNA does not have 5′–3′ polarity and a polyadenylated tail; thus, it is resistant to degradation by exonucleases [9]. Hence, circRNA is highly abundant, conserved, and stable and plays a key role in gene regulation by sponging microRNA (miRNA) [10, 11]. CircRNAs are closely related to the occurrence and development of central nervous system diseases [12], cardiovascular diseases [13], and cancer [14, 15], among many other diseases. In respiratory diseases, the research on circRNAs mainly focuses on the pathogenesis of lung cancer, and there are still few related studies on COPD. Altering the expression of miRNAs during the occurrence and development of diseases can regulate the pathogenesis of respiratory diseases [16]. Studies have proven that the levels of various miRNAs in the plasma of patients with COPD are significantly altered [17]. Therefore, circRNAs, as regulators of miRNAs, must participate in the pathogenesis of COPD by competing with endogenous RNA (ceRNA) networks.

The detection of biological factors in the plasma is convenient, and plasma collection is relatively easy to perform in the clinic. Therefore, in this study, we collected peripheral blood plasma specimens from patients with clinically very severe COPD and healthy control groups, analyzed the differential expression of circRNAs through microarray comparisons, constructed a ceRNA network, and performed verification using PCR technology. We explored the value of target circRNAs for the diagnosis and disease assessment of very severe COPD and provided directions for future research on signaling pathways related to pathogenesis.

## Methods

### Study population

Thirty patients with very severe COPD, thirty healthy control individuals, and fifteen patients with non-very severe COPD who were hospitalized at the Second People’s Hospital of Hefei from September 2021 to September 2022 were enrolled in this clinical study. Of the 30 patients with extremely severe COPD, 27 were male and 3 were female, aged 75 (71.8, 80.3) years, body mass index (BMI) 21.1 (18.8, 22.7) kg/m2, smoking history 40 (0.0, 40.0) pack-years. The 30 healthy control individuals that were randomly selected included 23 males and 7 females, aged 74 (67.8, 77.0) years, BMI 22.1 (19.9, 24.3) kg/m2, and smoking history 15 (0.0, 30.0) pack-years. There was no significant difference between the sex ratios, BMI, and age composition of the very severe COPD group and the healthy controls. All participants who met the guidelines for the diagnosis and treatment of COPD and with forced expiratory volume in one second (FEV1)/forced vital capacity of < 70% after inhalation of bronchodilators were included in the study. In addition, according to the joint guidelines of the American Thoracic Society and the European Respiratory Society on lung function, patients with an FEV1 as a percentage of the predicted value (FEV1% pred) less than 35% were classified as having extremely severe COPD. The exclusion criteria were as follows: (1) severe heart, liver, and kidney insufficiency; (2) past or current diagnosis of malignant tumors; (3) pregnancy; (4) other respiratory diseases affecting lung function; (5) patients with other infections; and (6) patients who were unwilling to cooperate or had an abnormal mental state. This study was approved by the medical ethics committee of the Second People’s Hospital of Hefei and conformed to the ethical guidelines of the Declaration of Helsinki. All enrolled patients and healthy controls signed informed consent for genetic analysis.

### RNA extraction and CircRNA microarray

An EDTA-K2 anticoagulant tube was used to collect 5 ml of fasting venous blood from all subjects in the morning. The tube was then centrifuged at 4 ℃ at 3000 rpm for 15 min, and the upper layer of plasma was transferred into a sterilized EP tube and stored in a freezer at -80 ℃. Blood samples from three patients with very severe COPD and three healthy controls were randomly selected. Total RNA was extracted from the samples using TRIzol reagent (Invitrogen, Carlsbad, CA, USA) following the manufacturer’s instructions. The concentrations of the RNA samples were determined by OD260 using a NanoDrop ND-1000 instrument. Sample labeling and array hybridization were carried out according to the manufacturer’s protocol. Total RNAs were digested with Rnase R (Epicentre, Madison, WI, USA) to eliminate linear RNAs and enrich circular RNAs. Then, the enriched circular RNAs were amplified and transcribed into fluorescent cRNA using a random priming method (Arraystar Super RNA Labeling Kit; Arraystar, Shanghai, China). The labeled cRNAs were hybridized onto the Arraystar Human circRNA Array V2 (8 × 15 K, Arraystar). After washing the slides, the arrays were scanned by using the Agilent Scanner G2505C.

### Data analysis

Quantile normalization of raw data and subsequent data processing were performed using the R software limma package. The differences were compared by calculating the fold change between the two groups of circRNAs. CircRNAs with fold change ≥ 1.3 and p-value ≤ 0.05 were selected as exhibiting significantly different expression patterns. For differentially expressed circRNAs (DEcircRNAs), we annotated the predicted target circRNA function using Gene Ontology (GO) and Kyoto Encyclopedia of Genes and Genomes (KEGG) signaling pathway analysis. We predicted circRNAs targeting miRNAs using TargetScan [18] and miRanda [19] and built ceRNA networks.

### Validation with RT-qPCR

RT-qPCR was performed on the RNA obtained from blood samples of the remaining 27 patients with COPD and 27 healthy controls, and the expression of related circRNAs was also verified in 15 patients with non-very severe COPD. The qRT-PCR method was as follows: according to the kit instructions, RNA was reverse-transcribed into cDNA using the riboSCRIPT Reverse Transcription Kit (C11027, RiboBio, Guangzhou, China), and qRT-PCR was performed using iQTM SYBR® Green Supermix (Bio-Rad, Hercules, CA, USA) with an LC480 fluorescent quantitative PCR instrument (Roche, Basel, Switzerland). The reaction conditions were as follows: 95 ℃ for 10 min, followed by 45 cycles of 95 ℃ for 10 s, 60 ℃ for 25 s, and 70 ℃ for 25 s. λ polyA (No.3789, TakaRa, Tokyo, Japan) was used as the external reference, and three complex wells were set up for each circRNA. The primer sequences used for circRNA analysis are shown in Table 1. The qRT-PCR results were analyzed by relative quantification using the 2^−ΔΔCt^ method.

**Table 1:** Primer sequences used for RT-qPCR

CircRNA	Forward primer (5′–3′)	Reverse primer (5′–3′)
hsa_circ_0008882	GGAATACCTTTCCTCACAGGAC	CTAGGCTGCCAATGGTGAG
hsa_circ_0089763	TTTAGTTGGGGCATTTATGTGA	CCTCAACCCAAAAAGGCATA
hsa_circ_0062683	GTCTCCCTGTCCAAGGCTCT	CCTTGGGCACTCTCATCTCT
hsa_circ_0077607	CTGCCCTTCACTTTGACCAG	GCTCCCAACTAGAAAGTATCTCTTCA

### Statistical analysis

Data were statistically analyzed using SPSS 25.0 and GraphPad Prism 9.0 software. Normally distributed data are expressed as (x ± s), and a *t*-test was used to examine differences between the two groups. Non-normally distributed data are expressed as interquartile ranges, and the Mann–Whitney U test was used for comparison between the two groups. Count data are expressed as n (%), and these were analyzed using the chi-square test. Statistical significance was set at P < 0.05.

## Results

### Subjects’ clinical characteristics

The clinical characteristics of 27 patients with very severe COPD and 15 patients with non-very severe COPD are shown in Table 2.

**Table 2:** Basic clinical information of the two chronic obstructive pulmonary disease (COPD) groups used in this study: Non-very severe COPD and Very severe COPD.

Variable		Non-very severe group	Very severe group	t/X2/z-value	P-value
Age (years)		75.0 (72.0, 79.0)	75.0 (71.0, 81.0)	-0.1	0.9
BMI (kg/m^2^)		21.6 (18.7, 24.5)	21.1 (18.6, 22.6)	-0.8	0.5
Male, n (%)		13 (86.7)	25 (92.6)	0.4	0.5
History of smoking (Pack-years)		30.0 (0.0, 40.0)	40.0 (20.0, 40.0)	-1.7	0.1
WBCs (10^9^/L)		6.7 (5.2, 8.1)	6.8 (5.4, 11.1)	-0.7	0.5
PCT (ng/mL)		0.0 (0.0, 0.1)	0.1 (0.0, 0.3)	-2.5	0.0*
CRP (mg/L)		3.4 (1.8, 19.7)	44.8 (5.9, 86.4)	-2.4	0.0*
FEV1 (L)		1.0 (0.8, 1.3)	0.6 (0.5, 0.8)	-3.8	0.0**
FVC (L)		1.8 ± 0.6	1.3 ± 0.4	3.7	0.0**
FEV1/FVC (%)		56.2 ± 6.1	52.7 ± 13.0	1.2	0.3
FEV1%pred (%)		41.1 (36.9,45.0)	25.9 (20.2, 31.7)	-5.3	0.0**

**Abbreviations**: BMI, body mass index; WBCs, white blood cells; PCT, procalcitonin; CRP, C reaction protein; FEV1, forced expiratory volume in 1 s; FEV1%pred, forced expiratory volume in 1 s as percentage of predicted value; FVC, forced vital capacity; * P-value < 0.05 ** P-value < 0.01. * p < 0.05 is considered to be significant

### Overview of CircRNA expression profiles

**Fig. 1:**
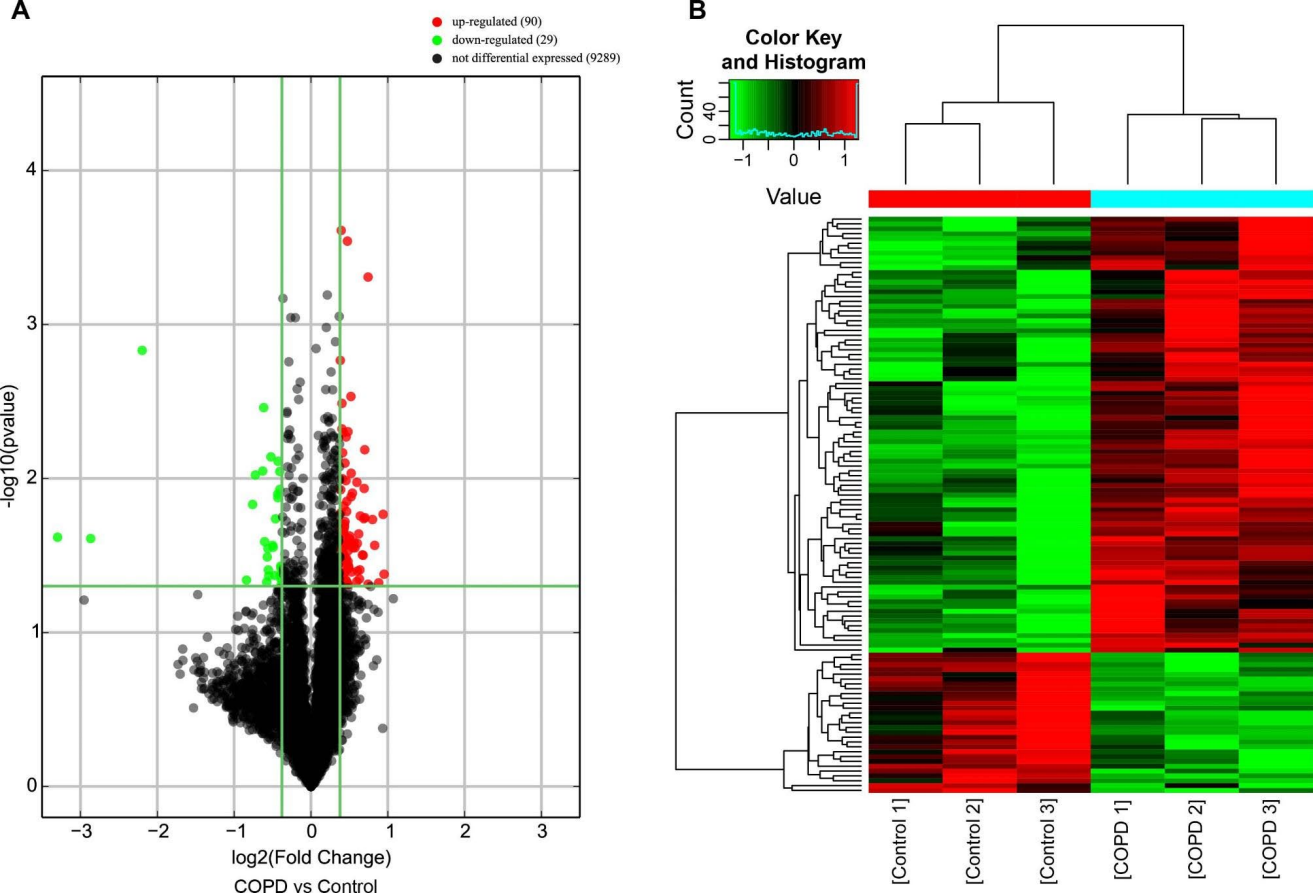
(**A**) Volcano plots were used to distinguish differentially expressed circRNAs. Red and green indicate up-regulation and down-regulation, respectively. (**B**) CircRNA heat map of significant difference in COPD. Each column corresponds to the expression profiles of one patient sample, and each row represents a circRNA. Red indicates higher expression level, green indicates lower expression level. **Abbreviations**: COPD, chronic obstructive pulmonary disease; CircRNA, circular RNA

Microarray technology was used to analyze the expression characteristics of circRNAs in the peripheral blood of three selected groups of patients with very severe COPD and three healthy control groups. The heatmap and volcano plot of DEcircRNAs are shown in Fig. 1. A total of 119 DEcircRNAs were obtained from 9289 human circRNAs. Among them, 90 circRNAs were upregulated, and 29 circRNAs were downregulated in the very severe COPD group compared with the control group. Table 3 lists the top ten upregulated and downregulated circRNAs.

**Table 3:** The top 10 circRNAs with the most significant difference in expression in the very severe COPD group

CircRNA	FC (abs)	P-value	circRNA_type	Circbase_id	Regulation	chrom
hsa_circRNA_089763	9.83	0.024	exonic	hsa_circ_0089763	Down	chrM
hsa_circRNA_089762	7.29	0.025	exonic	hsa_circ_0089762	Down	chrM
hsa_circRNA_008882	4.58	0.001	sense overlapping	hsa_circ_0008882	Down	chrM
hsa_circRNA_026129	1.79	0.046	sense overlapping	hsa_circ_0026129	Down	chr12
hsa_circRNA_051907	1.69	0.015	sense overlapping	hsa_circ_0051907	Down	chr19
hsa_circRNA_406281	1.65	0.009	intronic	-	Down	chr3
hsa_circRNA_001493	1.55	0.009	antisense	hsa_circ_0000851	Down	chr18
hsa_circRNA_091419	1.53	0.003	exonic	hsa_circ_0091419	Down	chrX
hsa_circRNA_004036	1.52	0.026	intergenic	hsa_circ_0004036	Down	chr9
hsa_circRNA_001595	1.49	0.047	antisense	hsa_circ_0001595	Down	chr6
hsa_circRNA_103188	1.93	0.042	exonic	hsa_circ_0062682	Up	chr22
hsa_circRNA_062683	1.92	0.017	exonic	hsa_circ_0062683	Up	chr22
hsa_circRNA_101506	1.84	0.048	exonic	hsa_circ_0003598	Up	chr15
hsa_circRNA_100837	1.78	0.027	exonic	hsa_circ_0022537	Up	chr11
hsa_circRNA_104172	1.74	0.018	exonic	hsa_circ_0077607	Up	chr6
hsa_circRNA_100100	1.67	0.049	exonic	hsa_circ_0010931	Up	chr1
hsa_circRNA_025249	1.67	0.000	exonic	hsa_circ_0025249	Up	chr12
hsa_circRNA_104220	1.63	0.018	exonic	hsa_circ_0006936	Up	chr6
hsa_circRNA_405540	1.62	0.007	intronic	-	Up	chr17
hsa_circRNA_090183	1.62	0.012	exonic	hsa_circ_0090183	Up	chrX

**Abbreviations**: circRNA, circular RNA ID, P-value, P-value calculated from t-test, FC (abs), Absolute fold change between two samples, CircRNA id, the circRNA ID in circBase (http://www.circbase.org/), Regulation, It depicts which sample has greater or lower intensity values with regards to other samples

### GO and KEGG pathway analyses

**Fig. 2:**
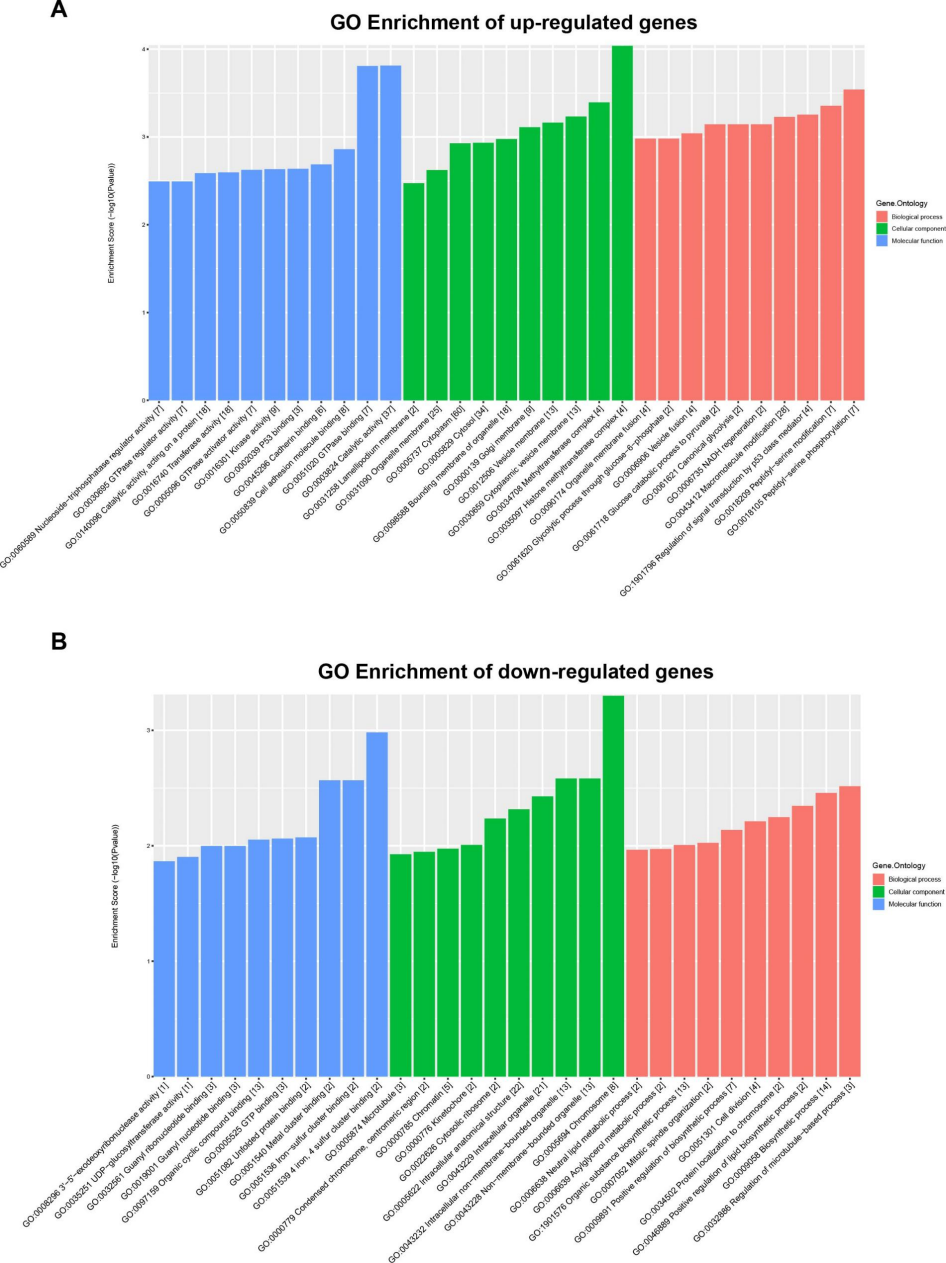
COPD differential circular RNA host gene GO function cluster map. **Abbreviations**: COPD, chronic obstructive pulmonary disease; GO, Gene Ontology; BP, biological process; CC, cellular component; MF, molecular function

**Fig. 3:**
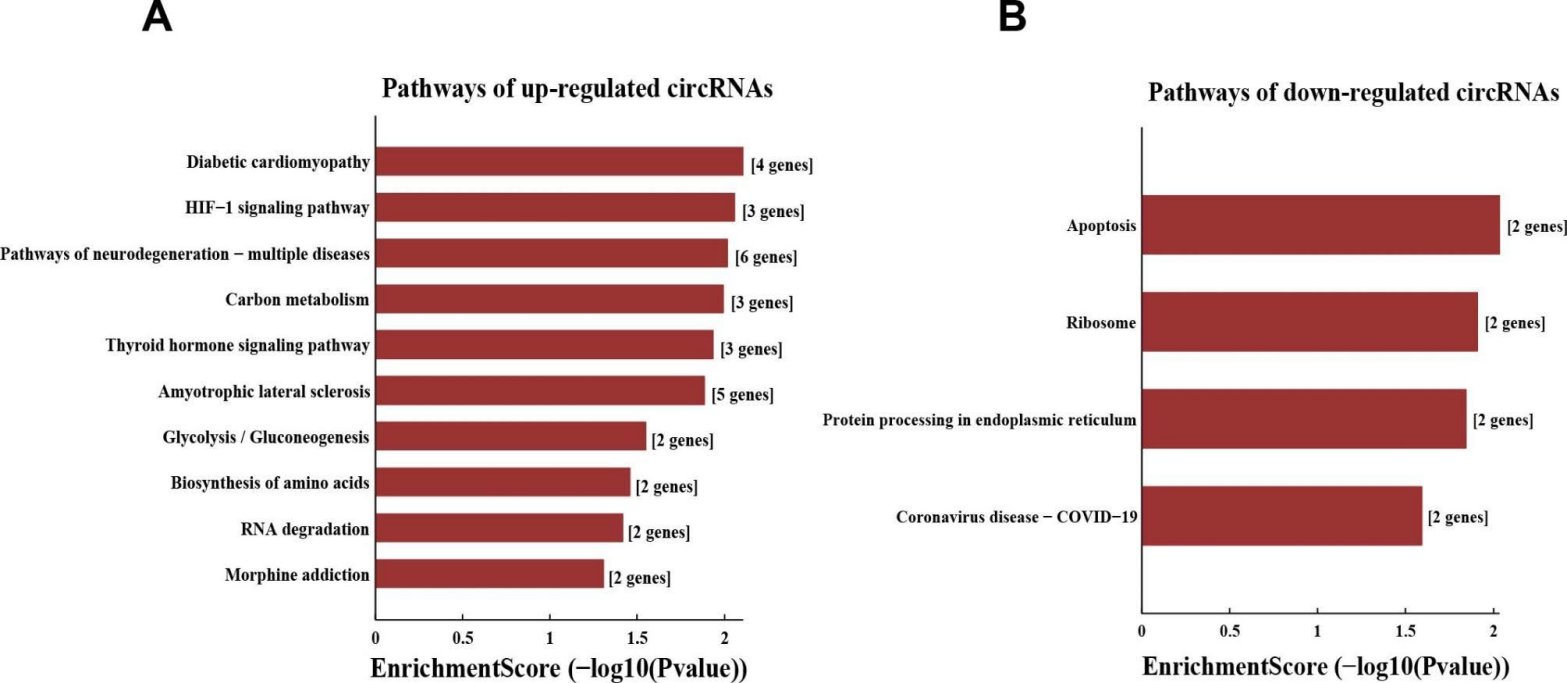
COPD differential circular RNA host gene KEGG signal pathway cluster map. **Abbreviations**: COPD, chronic obstructive pulmonary disease; KEGG, Kyoto Encyclopedia of Genes and Genomes

The DEcircRNAs were annotated using GO and KEGG signaling pathway analyses. The results of GO functional clustering are shown in Fig. 2. The three most significantly changed biological processes (BPs) were: regulation of signal transduction by p53 class mediator, peptidyl-serine modification, and peptidyl-serine phosphorylation. The three most significantly changed cell components (CCs) were: methyltransferase complex, histone methyltransferase complex, and chromosome. The three most significantly changed molecular functions (MFs) were: GTPase binding, catalytic activity, and 4 iron, 4 sulfur cluster binding. The results of KEGG signaling pathway clustering are shown in Fig. 3 [20]. The results show that the top three pathways with the most significant changes were: diabetic cardiomyopathy, HIF-1 signaling pathway, and apoptosis.

### CircRNAs-miRNAs interaction network

**Fig. 4:**
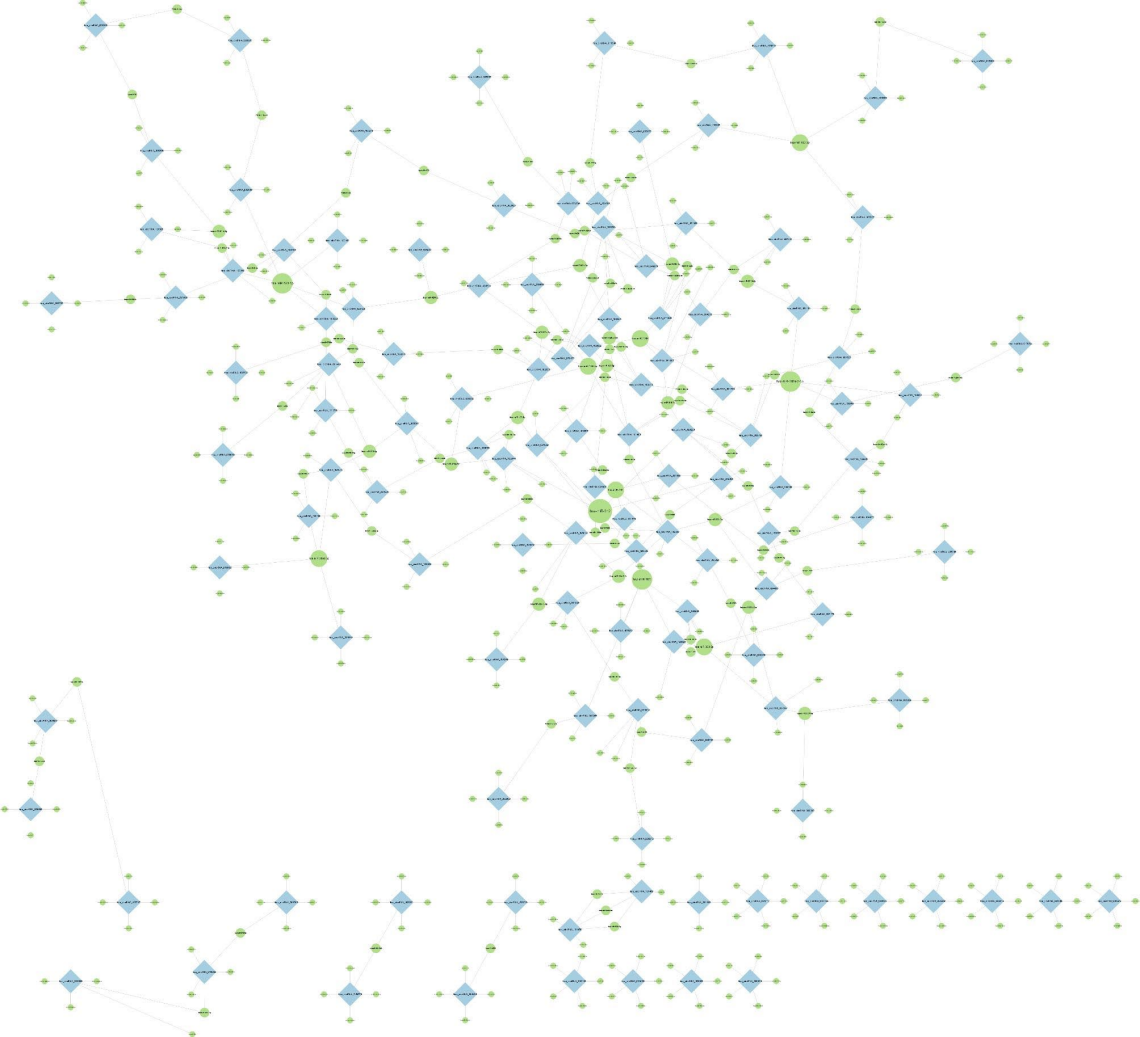
CircRNAs-miRNAs interaction network. CeRNA network of DEcircRNAs and miRNA interaction. Square nodes (blue) represent circRNAs, and circle nodes (green) represent miRNAs. The size of the node indicates the number of interactions. **Abbreviations**: CeRNA,competing with endogenous RNA; DEcircRNAs, differentially expressed circRNAs

To further explore the role of circRNA as an miRNA sponge, a circRNA-miRNA interaction network was constructed. Among them, hsa-miR-612, hsa-miR-593-5p, hsa-miR-765, and hsa-miR-103a-2-5p were the miRNAs regulated by more DEcircRNAs. The circRNA-miRNA interaction network constructed with Cytoscape_v3 6.1 is shown in Fig. 4.

### Validation of the DEcircRNAs

**Fig. 5:**
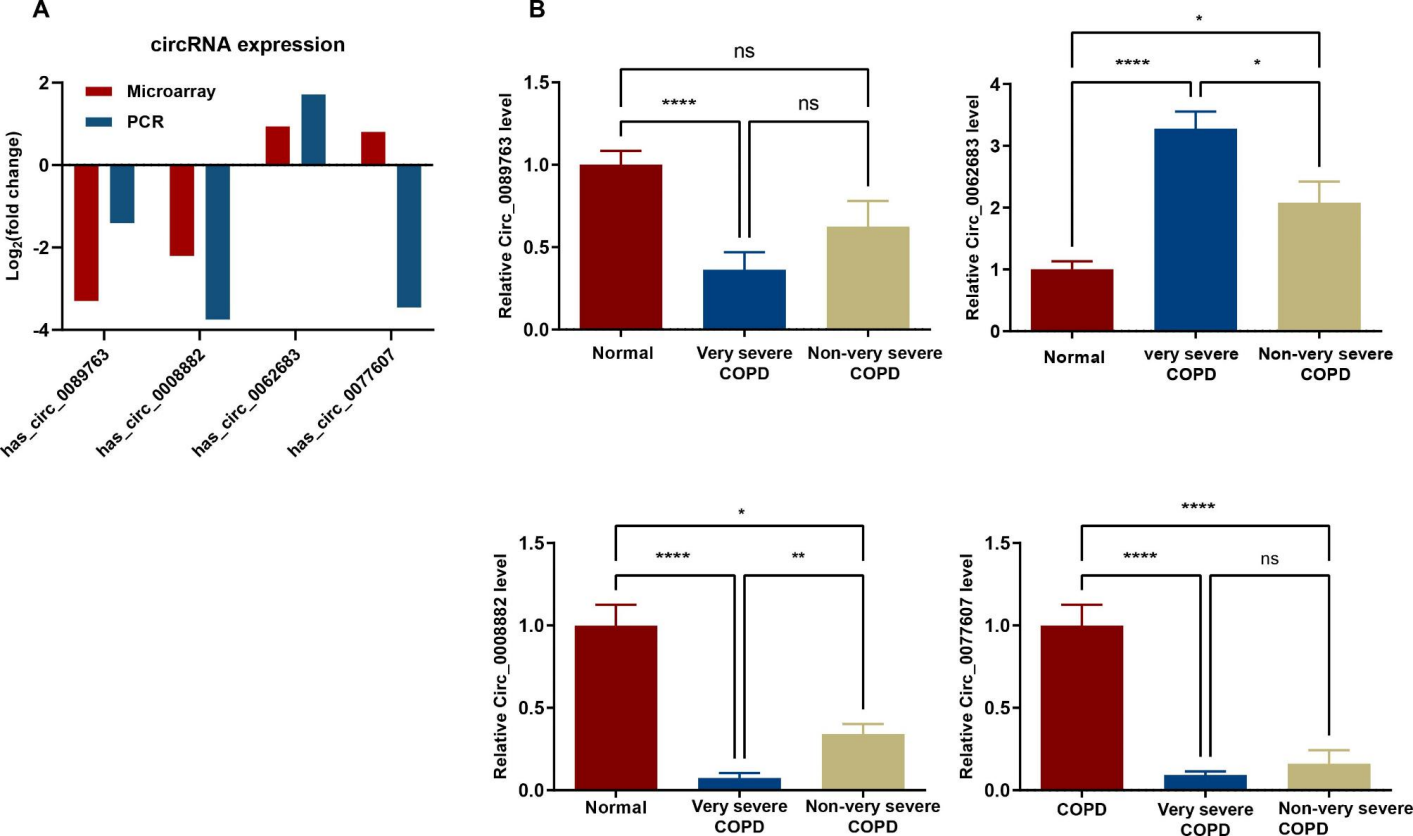
Validation of the DEcircRNAs by RT-qPCR. Four DEcircRNAs were validated by qRT-PCR, including two up‐regulated circRNAs and two down‐regulated circRNAs. (**A**) The heights of the columns in the chart represent the mean expression value of log2 fold changes. (**B**) The heights of the columns in the chart represent the relative expression (2^−ΔΔCT^). Data are mean ± SEM. *P-value < 0.05. **P-value < 0.01. ****P-value < 0.0001. ns, no significance. DEcircRNAs, differentially expressed circRNAs.

Four DEcircRNAs were selected and validated by qRT-PCR in the remaining 27 patients with very severe COPD and 27 healthy controls. We also validated the results using 15 patients with COPD that is not very severe to investigate whether these differentially expressed genes were also expressed in these patients and their value in differentiating very severe COPD from eother degrees of COPD severity. The results of qRT-PCR validation are shown in Fig. 5. According to the results, hsa_circ_0062683, hsa_circ_0089763, and hsa_circ_0008882 expression showed consistency with the predicted results; however, the hsa_circ_0077607 expression showed an opposite result to the microarray results. Among them, hsa_circ_0062683 was significantly upregulated, and hsa_circ_0089763 and hsa_circ_0008882 were significantly downregulated. Simultaneously, hsa_circ_0062683 and hsa_circ_0008882 also showed different expressions in patients with non-very severe COPD compared with that in the healthy control group (P < 0.05). There were also significant differences between very severe COPD and non-very severe COPD (P < 0.05).

### Correlation analyses of the circRNAs level with clinical data

Three circRNAs consistent with the predicted results were selected and their correlation with clinical indicators in COPD patients was compared. The relationship between the circRNAs expression and clinical data is shown in Table 4. Correlation analyses showed that the hsa_circ_0062683 expression level in COPD patients was positively correlated with age and negatively correlated with FEV1%pred. hsa_circ_0089763 and hsa_circ_0008882 were negatively correlated with white blood cells (WBCs) and positively correlated with FEV1% pred. In addition, hsa_circ_0008882 also negatively correlated with C reaction protein (CRP).

**Table 4:** Correlation analyses of the circRNAs level with clinical data

Variable	hsa_circ_0062683	hsa_circ_0089763	hsa_circ_0008882
WBCs	ns	r = -0.390*	r = -0.562**
FEV1%pred	r = -0.415**	r = 0.405**	r = 0.570**
BMI	ns	ns	ns
Age	r = 0.328*	ns	ns
CRP	ns	ns	r = -0.423**

**Abbreviations**: BMI, body mass index; WBCs, white blood cells; CRP, C reaction protein; FEV1%pred, forced expiratory volume in 1 s as percentage of predicted value; ns, no significance. * P-value < 0.05 ** P-value < 0.01. * p < 0.05 is considered to be significant

### ROC curves for the DEcircRNAs

**Fig. 6:**
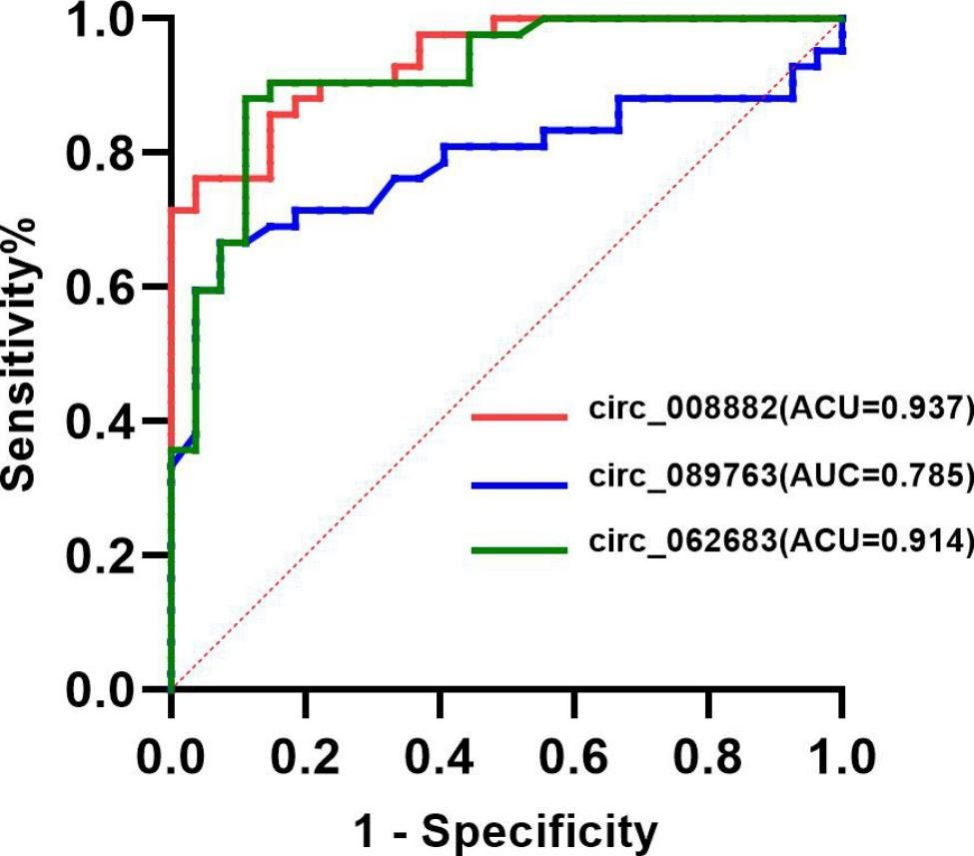
Receiver operating characteristic curves for hsa_circ_0062683, hsa_circ_0089763, and hsa_circ_0008882. AUC, area under the curve

The predictive value of hsa_circ_0062683, hsa_circ_0089763, and hsa_circ_0008882 for COPD is shown in Fig. 6. Receiver operating characteristic (ROC) curves analysis showed that hsa_circ_0062683 (AUC = 0.914, sensitivity 88.1%, specificity 88.9%), hsa_circ_0089763 (AUC = 0.785, sensitivity 66.7%, specificity 92.6%), and hsa_circ_0008882 (AUC = 0.937, sensitivity 76.2%, specificity 96.3%) were valuable in the clinical diagnosis of COPD.

## Discussion

COPD is clinically manifested by progressive irreversible airflow limitation and lung parenchymal damage [21], which is largely attributed to the chronic inflammatory response of the airways and lungs to harmful particles or gases [2]. As a progressive inflammatory disease, there is still controversy regarding which aspects of COPD are preventable and treatable [22]; thus, further research is needed. CircRNAs play a crucial role in gene regulation by sponging miRNAs or proteins [23]. The unique structural conformation of circRNAs and their stable characteristics give them the potential to be used as biomarkers [24]. Numerous studies have shown that circRNAs play an important role in the occurrence and development of lung diseases. For example, CircFOXO3 plays a key role in the pathological remodeling of inflammatory processes induced by cigarette smoke exposure (CSE) and may become a good target for the treatment of inflammatory diseases [25]. Circ_0061052 regulates miR-515-5p through the FoxC1/Snail regulatory axis, thereby participating in CSE-induced epithelial-mesenchymal transition and airway remodeling in COPD [26]. Xue et al. found that circ_0006872 promotes apoptosis, inflammation, and oxidative stress in human pulmonary microvascular endothelial cells (PMVECs) through the miR-145-5p/NF-κB axis [27]. Through the identification of circRNA expression in patients’ peripheral blood core solo cells, it was found that circRNAs may be involved in the development of COPD by affecting immune balance [28].

Environmental exposures such as CSE and air pollutants can differentiate the expression profiles of miRNAs in patients with COPD from those in healthy individuals [29–31]. CircRNAs, as important regulators of miRNAs, definitely affect these differentially expressed miRNAs and play a role in the key signaling pathways involved in the pathogenesis of COPD. Studies have shown that the expression differences of miRNAs are related to the severity of emphysema in patients with COPD [32]. Therefore, genome-wide DEcircRNAs in patients with very severe COPD and healthy people have research significance. In this study, difference analysis of plasma circRNA expression profiles between patients with very severe COPD and healthy control groups was carried out for the first time. A total of 119 DEcircRNAs were obtained from 9289 human circRNAs. Among them, 90 circRNAs were upregulated, and 29 circRNAs were downregulated in the very severe COPD group compared with those in the control group. We verified 4 DEcircRNAs using qRT-PCR, and the results revealed that the expression of hsa_circ_0062683, hsa_circ_0089763, and hsa_circ_0008882 exhibited the same pattern as the predicted results. The ROC curve also verified the value of these circRNAs in clinical diagnosis. These circRNAs may be involved in the pathogenesis of COPD. In previous reports, overexpression of hsa_circ_0008882 inhibited the release of cytokines after copper exposure, thereby playing a role in the regulation of inflammatory responses related to chronic respiratory diseases [33]. Because of the characteristics of sequence lengths and partial cytoplasmic localization, hsa_circ_0089763 acts as a powerful miRNA sponge [34]. hsa_circ_0062683 is proposed for the first time in this study to be involved in COPD and may be associated with clinical indicators such as age. Some studies have found that the density of neutrophils in the lung parenchyma of patients with very severe COPD is significantly higher than that of other COPD patients [35]. Correlation studies also show the correlation between hsa_circ_0008882 and WBCs and CRP. The relationship of hsa_circ_0008882 with inflammation regulation and its impact on the onset of very severe COPD warrants further study. Furthermore, compared with that in the healthy control group, hsa_circ_0062683 and hsa_circ_0008882 also showed different expression patterns in patients with non-very severe COPD. FEV1% pred has been used clinically to distinguish the degree of airflow limitation in patients with COPD, and correlation analysis suggests a correlation between these circRNAs and FEV1% pred. Thus, they may have value as biomarkers for the diagnosis of COPD and in assessing the disease severity.

GO enrichment analysis of DEcircRNAs was also performed and revealed that the BP and CC processes mainly involved in the pathogenesis of very severe COPD were the regulation of signal transduction by the p53 class mediator and methyltransferase complex. The most apparent change in MF was GTPase binding, which is related to the reduction of lung injury, fibrosis, and alveolar epithelial cell death [36]. It also plays a vital role in regulating the permeability of endothelial cells [37]. In addition, it is related to important regulators of antiviral defense mechanisms [38]. Related circRNAs may affect the occurrence and development of very severe COPD through these functions. Diabetic cardiomyopathy and HIF-1 signaling pathway are significantly upregulated in KEGG signaling pathway changes. The HIF-1 signaling pathway has been studied in a large number of related mechanisms such as cell injury, oxidative stress, and hypoxia [39]. In addition, miRNAs play a key role in the hypoxia response pathway [40]. Apoptosis and Ribosome are significantly down-regulated in pathway changes. These findings provide new clues for the study of COPD. Studies have shown that COPD is related to the CSE-induced apoptosis of PMVECs [41]. Therefore, it is inferred that these DEcircRNAs regulate miRNAs through the aforementioned pathways to play a role in the pathogenesis of COPD.

By constructing the circRNA-miRNA interaction network, it was found that hsa-miR-612, hsa-miR-593-5p, hsa-miR-765, and hsa-miR-103a-2-5p are the miRNAs regulated by more DEcircRNAs. Studies have shown that hsa-miR-612 may play a key role in the regulation of endothelial cell angiogenesis under hypoxia [42]. The construction of an in vitro airway epithelial model revealed abundant hsa-miR-612, which is involved in regulating the key pathways of airway differentiation and remodeling [43]. In addition, surfactant protein A (SP-A) can regulate the function of a variety of immune cells, whereas hsa-miR-612 may promote susceptibility to lung diseases by inhibiting the gene encoding SP-A [44]. These results suggest that DEcircRNAs that regulate hsa-miR-612, such as hsa_circ_0007677 and hsa_circ_0001952, may play a role in the development of very severe COPD through hypoxia or mechanisms that regulate immune cells. In the future, the functions of candidate DEcircRNAs require further investigation.

This experiment had some limitations. First, there were deficiencies due to patient sample sizes and this being a single-center study. Of the four cicrRNAs verified in this study, one showed the opposite direction. Microarray as a gene screening technology, in the future, the sample sizes of patients with COPD and healthy control groups need to be further expanded, and numerous clinical samples need to be repeatedly screened and verified. Second, patients with very severe COPD often exhibit influencing factors such as advanced age and combined underlying diseases. In addition, there are many people with a history of smoking in COPD patients. Whether smoking as a potential confounding factor has a certain impact on the expression results is worth further exploring. Hence, more verification of relevant targets in combination with basic research in the future is required. Through experimental verification of core genes, the occurrence and development of related diseases can be more clearly understood.

## Conclusion

In this study, we compared the expression of circRNAs in patients with very severe COPD and a healthy control group by the microarray method and performed verification using qRT-PCR for bioinformatic analysis. The results confirmed that compared with that in the healthy control group, hsa_circ_0062683 of patients with very severe COPD was significantly upregulated, whereas the hsa_circ_0089763 and hsa_circ_0008882 were significantly downregulated. This difference indicates that plasma circRNAs may play a role in the diagnosis and disease assessment of very severe COPD. DEcircRNAs may participate in the development of COPD through hypoxia or regulation of a variety of immune cells, which requires more research in the future.

## Data Availability

The datasets generated and analysed during the current study are available in the GEO repository, https://www.ncbi.nlm.nih.gov/geo/query/acc.cgi?acc=GSE221812.

## Abbreviations

BMI: Body mass index
BP: Biological process
CC: Cellular component
ceRNA: Competing endogenous RNA
circular RNA: CircRNA
COPD: Chronic Obstructive Pulmonary Disease
CRP: C reaction protein
CSE: Cigarette smoke exposure
DEcircRNAs: Differentially expressed circRNAs
FEV1%pred: Forced expiratory volume in 1 s as percentage of predicted value
FEV1: Forced expiratory volume in 1 s
FVC: Forced vital capacity
GO: Gene Ontology
KEGG: Kyoto Encyclopedia of Genes and Genomes
MF: Molecular function
miRNA: MicroRNA
PCT: Procalcitonin
PMVECs: Pulmonary microvascular endothelial cells
qRT-PCR: Quantitative real-time polymerase chain Reaction
ROC: Receiver operating characteristic
SP-A: Surfactant protein A
WBCs: White blood cells
